# Visceral Adiposity Thresholds for Cardiovascular Risk Stratification: A Simplified Biomarker‐Driven Model

**DOI:** 10.1002/oby.24367

**Published:** 2025-08-18

**Authors:** Tangmeng Guo, Ping He, Weilin Lu, Lili Huang, Chengyun Liu, Bei Cheng, Yuanyuan Zhang, Qi Zhang, Yanxu Chen, Minghao Liu, Peien Zhou, Junxi Liu, Xinchun Gu, Zhengyang Sun, Qiang Zhang, Sihao Xiao

**Affiliations:** ^1^ Department of Geriatrics, Union Hospital, Tongji Medical College Huazhong University of Science and Technology Wuhan China; ^2^ Institute of Gerontology, Union Hospital, Tongji Medical College Huazhong University of Science and Technology Wuhan China; ^3^ Division of Cardiovascular Medicine, John Radcliffe Hospital University of Oxford Oxford UK; ^4^ Department of Clinical Laboratory Medicine, People's Hospital of Dongxihu District Wuhan City and Union Dongxihu Hospital Huazhong University of Science and Technology Wuhan China; ^5^ Echocardiography and Vascular Ultrasound Centre, The First Affiliated Hospital Zhejiang University School of Medicine Hangzhou China; ^6^ Department of Radiology Huashan Hospital Fudan University Shanghai China; ^7^ Department of Organ Transplantation, The First Affiliated Hospital of Sun Yat‐Sen University Guangzhou China; ^8^ Department of Computer Science University of Oxford Oxford UK; ^9^ Nuffield Department of Population Health, Oxford Population Health University of Oxford Oxford UK; ^10^ Nuffield Department of Primary Care Health Sciences University of Oxford Oxford UK; ^11^ Oxford Centre for Clinical Magnetic Resonance Research, Division of Cardiovascular Medicine, John Radcliffe Hospital University of Oxford Oxford UK

## Abstract

**Objective:**

VAT volume is a critical determinant of cardiometabolic risk, yet population‐specific thresholds and accessible predictive tools remain undefined.

**Methods:**

Using US National Health and Nutrition Examination Survey (NHANES) data, we analyzed dual‐energy x‐ray absorptiometry (DXA)‐derived VAT volume. Threshold effects and sex/ethnicity interactions were evaluated via multivariable regression, while LASSO regularization and ROC analyses identified a simplified predictive model.

**Results:**

A VAT volume threshold of 327.0 cm^3^ stratified CVD risk, distinguishing compensatory from pathological adiposity. The high‐VAT group exhibited elevated CVD prevalence (2.41% vs. 1.12%, *p* = 0.016), metabolic dysregulation, and socioeconomic disparities. Males showed higher risk thresholds than females (387.5 vs. 312.0 cm^3^, *p*‐interaction = 0.029). Non‐Hispanic White participants and multiracial groups exhibited abrupt risk escalation above 399.5 and 270.0 cm^3^ (aOR = 1.08–1.12, *p* < 0.001), absent in non‐Hispanic Black individuals and Hispanic individuals. A tri‐biomarker model (waist circumference + triglycerides + apolipoprotein B) achieved near‐equivalent accuracy to DXA‐based VAT quantification (AUC = 0.821 vs. 0.819, *p* = 0.66), with high sensitivity (80.95% vs. 69.05%) and cost‐effectiveness.

**Conclusions:**

This study establishes the first sex‐specific and ethnicity‐specific VAT thresholds for CVD risk stratification and provides a clinically actionable tool for visceral adiposity screening.

**Study Importance**

What is already known?

- Current clinical practice relies on BMI, calculated as weight in kilograms divided by height in meters squared, for obesity classification.
- This metric fails to capture critical pathophysiological distinctions in regional adiposity deposition, conflating lean mass with fat mass and disregarding visceral adipose tissue (VAT) distribution patterns.

What does this study add?

- Sex‐ and ethnicity‐specific VAT thresholds: for the first time, VAT volume thresholds were established to stratify cardiovascular disease (CVD) risk, with distinct thresholds for males (387.5 cm^3^) and females (312.0 cm^3^). Notably, risk escalation thresholds varied by ethnicity, such as 399.5 cm^3^ for non‐Hispanic White participants and 270.0 cm^3^ for multiracial groups, while no clear thresholds were observed in non‐Hispanic Black participants and Hispanic participants.
- Nonlinear CVD risk relationship: VAT volume demonstrated a nonlinear association with CVD risk, challenging the assumption of linear risk progression. Pathological adiposity (> 327.0 cm^3^) was linked to a doubling of CVD prevalence (2.41% vs. 1.12%, *p* = 0.016).
- Tri‐biomarker predictive model: a simplified clinical tool combining waist circumference, triglycerides, and apolipoprotein B achieved diagnostic accuracy comparable to dual‐energy x‐ray absorptiometry‐based VAT quantification (AUC = 0.821 vs. 0.819, *p* = 0.66), offering a cost‐effective alternative to imaging.

## 1 Introduction

Overweight and obesity constitute a critical global health challenge, threatening to reverse decades of progress in population health outcomes [1]. Projections estimate that by 2050, approximately 3.8 billion adults worldwide will live with overweight or obesity—representing over half of the projected global adult population—with China, India, and the United States remaining predominant contributors to this burden [2].

Current clinical practice relies on body mass index (BMI), calculated as weight in kilograms divided by height in meters squared, for obesity classification. However, this metric fails to capture critical pathophysiological distinctions in regional adiposity deposition, conflating lean mass with fat mass and disregarding visceral adipose tissue (VAT) distribution patterns [3]. Consequently, BMI‐based diagnoses may misclassify cardiometabolic risk, either underestimating VAT‐driven pathology in normal‐weight individuals or overestimating risk in muscular phenotypes.

Emerging evidence suggests that integrating waist circumference (WC) with biochemical markers—particularly plasma triglycerides (TG) and apolipoprotein B (ApoB)—may enhance risk stratification accuracy by quantifying both abdominal adiposity topography and VAT‐associated dyslipidemia [4]. This combined approach could improve the identification of individuals with ectopic fat deposition and elevated cardiovascular disease (CVD) susceptibility.

This study therefore aims to:

- Establish sex‐ and ethnicity‐specific VAT volume thresholds for CVD risk stratification.
- Evaluate the diagnostic performance of WC combined with lipid biomarkers in predicting VAT accumulation.
- Develop a simplified clinical model for visceral adiposity assessment that balances accuracy with practicality.
- By addressing critical gaps in obesity phenotyping, advance precision medicine approaches for CVD prevention in diverse populations.

## 2 Methods

### 2.1 Study Design and Participants

This cross‐sectional analysis included 8552 US adults aged ≥ 20 years from four cycles (2011–2018) of the National Health and Nutrition Examination Survey (NHANES), a nationally representative dataset employing a stratified, multistage probability sampling design [5]. Participants underwent dual‐energy x‐ray absorptiometry (DXA) for VAT quantification. NHANES protocols integrate demographic, socioeconomic, clinical, and laboratory data collected via structured interviews, physical examinations, and biospecimen analyses. The National Center for Health Statistics Institutional Review Board approved all procedures, and written informed consent was obtained from all participants.

### 2.2 Data Collection

Data on age, sex, race, ethnicity, VAT, BMI, WC, education, smoking history, physical activity, hypertension, hypercholesterolemia, diabetes, hemoglobin A1c, TG, ApoB, high‐density lipoprotein cholesterol (HDL‐C), low‐density lipoprotein cholesterol (LDL‐C), and high‐sensitivity C‐reactive protein (Hs‐CRP) were collected. Smoking history was defined as self‐report of smoking at least 100 cigarettes ever. A self‐reported history of coronary heart disease, stroke, hypertension, hypercholesterolemia, and diabetes was defined as having ever been diagnosed with them. CVD includes coronary heart disease and stroke. The “self‐reported race and ethnicity” categories comprise the following: Hispanic individuals (Mexican American and other Hispanic individuals), non‐Hispanic White individuals, non‐Hispanic Black individuals, and other races (including multiracial individuals).

Weight, height, and WC were measured during the physical examination, and BMI was calculated as weight in kilograms divided by height in meters squared. Blood samples were collected at the mobile examination center, stored at −20°C, and sent to the central laboratories for the determination using standard methods. VAT (cm^3^) was measured by DXA in the NHANES mobile examination center.

### 2.3 Statistical Analysis

Continuous variables are presented as mean ± SD and categorical variables as percentages. Optimal VAT thresholds were identified via likelihood ratio tests. Predictor selection for VAT volume estimation employed LASSO regression with lambda.1se (optimal regularization parameter balancing model simplicity and overfitting risk). Diagnostic performance of WC, TG, and ApoB—individually and combined—was assessed via receiver operating characteristic (ROC) curves, with area under the curve (AUC), sensitivity, specificity, and likelihood ratios calculated. All analyses were performed using EmpowerStats (v4.1, X&Y Solutions) and R (v4.3.2, R Foundation), with two‐tailed *p* < 0.05 denoting statistical significance.

## 3 Results

Table 1 summarizes the demographic and clinical characteristics of NHANES participants stratified by the VAT volume threshold of 327.0 cm^3^, derived from threshold effect analysis for CVD risk (Table 2). The cohort was dichotomized into low‐VAT (≤ 327.0 cm^3^, *n* = 984) and high‐VAT (> 327.0 cm^3^, *n* = 2327) groups. The high‐VAT group demonstrated a markedly higher mean VAT volume compared to the low‐VAT group (650.31 ± 259.59 vs. 227.25 ± 64.20 cm^3^, *p* < 0.001). Consistent with metabolic syndrome profiles, the high‐VAT group exhibited significantly elevated CVD risk markers: older age (mean difference: +9.0 years, 41.53 ± 10.77 vs. 32.55 ± 10.64 years, *p* < 0.001), greater WC (+21.4 cm, 10.32 ± 1.45 vs. 8.18 ± 0.99 cm, *p* < 0.001), higher BMI (30.83 ± 6.48 vs. 23.32 ± 4.16 kg/m^2^, *p* < 0.001), and adverse lipid profiles including TG (1.52 ± 1.16 vs. 0.87 ± 0.50 mmol/L, *p* < 0.001) and ApoB (0.96 ± 0.25 vs. 0.77 ± 0.20 g/L, *p* < 0.001).

**TABLE 1:** Baseline characteristics and CVD risk stratification by visceral adipose tissue volume thresholds in the study cohort.

Characteristics	VAT volume(cm^3^)		*p*
	≤ 327.0	> 327.0	
Age, years	32.55 ± 10.64	41.53 ± 10.77	< 0.001
Sex			< 0.001
Male	455 (46.24%)	1316 (56.55%)	
Female	529 (53.76%)	1011 (43.45%)	
Race/ethnicity			< 0.001
Non‐Hispanic White	362 (36.79%)	912 (39.19%)	
Non‐Hispanic Black	252 (25.61%)	432 (18.56%)	
Hispanic	147 (14.94%)	637 (27.37%)	
Other (including multiracial)	223 (22.66%)	346 (14.87%)	
VAT volume, cm^3^	227.25 ± 64.20	650.31 ± 259.59	< 0.001
WC, cm	81.8 ± 9.9	103.2 ± 14.5	< 0.001
BMI, kg/m^2^	23.32 ± 4.16	30.83 ± 6.48	< 0.001
TG, mmol/L	0.87 ± 0.50	1.52 ± 1.16	< 0.001
ApoB, g/L	0.77 ± 0.20	0.96 ± 0.25	< 0.001
Smoking status	360 (36.59%)	1001 (43.02%)	< 0.001
Physical activity, MET‐h/week	117.33 ± 516.60	90.31 ± 192.84	0.029
Hypertension	109 (11.08%)	683 (29.35%)	< 0.001
Hypercholesterolemia	106 (10.77%)	686 (29.48%)	< 0.001
Diabetes	13 (1.32%)	214 (9.20%)	< 0.001
CVD	11 (1.12%)	56 (2.41%)	0.016
Education level			< 0.001
Less than high school graduate	132 (13.41%)	453 (19.47%)	
High school graduate or GED	175 (17.78%)	517 (22.22%)	
Some college or above	677 (68.80%)	1357 (58.32%)	

*Note*: Values are mean ± SD/*N*(%).

Abbreviations: CVD, cardiovascular disease; GED, general equivalency diploma; TG, triglycerides; VAT, visceral adipose tissue; WC, waist circumference.

**TABLE 2:** Sex‐specific threshold effects of visceral adipose tissue volume on cardiovascular risk: stratified nonlinear association analysis.^a^

Sex	Male	Female	Total
Model 1			*p*‐interaction: 0.029
A linear effect	1.07 (1.04, 1.09) < 0.0001	1.04 (1.01, 1.07) 0.0138	1.05 (1.03, 1.07) < 0.0001
Model 2			*p*‐interaction: 0.214
Kink point (K, cm^3^)	387.5	312.0	327.0
< K segment effect 1	0.87 (0.71, 1.07) 0.1925	0.91 (0.75, 1.12) 0.3732	0.87 (0.74, 1.01) 0.0754
> K segment effect 2	1.08 (1.05, 1.11) < 0.0001	1.05 (1.01, 1.08) 0.0053	1.06 (1.04, 1.09) < 0.0001
Difference in effect between 2 and 1	1.24 (0.99, 1.54) 0.0564	1.15 (0.93, 1.42) 0.2125	1.23 (1.04, 1.45) 0.0150
Predicted value of the kink point prescription equation	−4.25 (−4.53, −3.96)	−4.24 (−4.55, −3.93)	−4.31 (−4.52, −4.10)
Log‐likelihood ratio test	0.068	0.227	0.021

*Note*: Values are β (95% CI) *p* value/OR (95% CI) *p* value.

^a^ β or OR is calculated based on the transformed VAT volume. The transformed VAT volume was obtained by multiplying the raw data by 0.02. Adjust for: age, race, smoking status.

Lifestyle and clinical risk disparities were pronounced: the high‐VAT group had higher smoking rates (43.02% vs. 36.59%, *p* < 0.001), reduced physical activity (90.31 ± 192.84 vs. 117.33 ± 516.60 MET‐min/week, *p* = 0.029), and greater prevalence of hypertension (29.35% vs. 11.08%, *p* < 0.001), hypercholesterolemia (29.48% vs. 10.77%, *p* < 0.001), diabetes (9.20% vs. 1.32%, *p* < 0.001), and CVD (2.41% vs. 1.12%, *p* = 0.016).

Notable sociodemographic differences included a higher proportion of males (56.55% vs. 46.24%, *p* < 0.001) and Hispanic individuals (27.37% vs. 14.94%, *p* < 0.001) and lower attainment of tertiary education (58.32% vs. 68.80%, *p* < 0.001) in the high‐VAT group. These findings collectively characterize high visceral adiposity as a hallmark of metabolic dysregulation, disproportionately affecting males, Hispanic populations, and individuals with lower socioeconomic status. The data robustly validate VAT volume as a pivotal biomarker for obesity‐related cardiometabolic risk stratification.

Table 2 presents the threshold effect analysis of VAT volume on CVD risk, stratified by sex. Each unit increase in transformed VAT volume was associated with a 5% elevated CVD risk (adjusted odds ratio [aOR] = 1.05, 95% confidence interval [CI] = 1.03–1.07, *p* < 0.0001). Sex‐stratified analysis revealed significant heterogeneity: males exhibited a 7% increased CVD risk per transformed VAT unit (aOR = 1.07, 95% CI = 1.04–1.09, *p* < 0.0001), while females demonstrated a 4% risk increment (aOR = 1.04, 95% CI = 1.01–1.07, *p* = 0.0138). A significant sex interaction was observed (*p*‐interaction = 0.029), confirming stronger VAT‐associated CVD susceptibility in males.

Nonlinear threshold analysis identified sex‐specific inflection points:

- Males: below the threshold (K = 387.5 cm^3^), VAT showed no significant CVD association (aOR = 0.87, 95% CI = 0.71–1.07, *p* = 0.1925). Above K, each VAT unit conferred an 8% risk surge (aOR = 1.08, 95% CI = 1.05–1.11, *p* < 0.0001).
- Females: Subthreshold VAT volumes (K = 312.0 cm^3^) showed null effects (aOR = 0.91, 95% CI = 0.75–1.12, *p* = 0.3732), while suprathreshold levels triggered a 5% risk elevation (aOR = 1.05, 95% CI = 1.01–1.08, *p* = 0.0053).

These results delineate a sexually dimorphic risk architecture: males require higher VAT thresholds to initiate pathological risk escalation, whereas females exhibit earlier but attenuated risk progression. The differential thresholds (ΔK = 75.5 cm^3^) and effect magnitudes (male aOR = 1.08 vs. female aOR = 1.05) suggest fundamental biological disparities in VAT‐driven cardiometabolic pathophysiology.

Table 3 delineates race/ethnicity‐specific threshold effects in the association between VAT volume and CVD. In Model 1 (linear effect analysis), non‐Hispanic White individuals exhibited a 7% increased CVD risk per transformed VAT increment (aOR = 1.07, 95% CI = 1.04–1.10, *p* < 0.0001), while the group including “other races” (including multiracial) demonstrated a 10% risk elevation (aOR = 1.10, 95% CI = 1.04–1.16, *p* = 0.0005). No significant associations were observed for non‐Hispanic Black participants or Hispanic participants (*p* > 0.05). The race‐VAT interaction approached statistical significance (*p*‐interaction = 0.051), suggesting potential ethnic heterogeneity in VAT‐driven CVD risk.

**TABLE 3:** Racial/ethnic heterogeneity in visceral adipose tissue volume thresholds for CVD risk: ethnicity‐stratified threshold effect analysis.^a^

Race/ethnicity	Non‐Hispanic White	Non‐Hispanic Black	Hispanic	Other races	Total
Model 1					*p*‐interaction: 0.051
A linear effect	1.07 (1.04, 1.10) < 0.0001	1.02 (0.98, 1.07) 0.2460	1.02 (0.97, 1.07) 0.4548	1.10 (1.04, 1.16) 0.0005	1.05 (1.03, 1.07) < 0.0001
Model 2					*p*‐interaction: 0.213
Kink point (K)	399.5	155.0	274.5	270.0	327.0
< K segment effect 1	0.92 (0.76, 1.11) 0.3832	0.52 (0.18, 1.55) 0.2424	0.70 (0.37, 1.34) 0.2811	0.63 (0.37, 1.07) 0.0899	0.87 (0.74, 1.01) 0.0754
> K segment effect 2	1.08 (1.05, 1.11) < 0.0001	1.03 (0.99, 1.07) 0.1806	1.03 (0.98, 1.08) 0.3061	1.12 (1.06, 1.19) < 0.0001	1.06 (1.04, 1.09) < 0.0001
Difference in effect between 2 and 1	1.17 (0.96, 1.43) 0.1185	1.97 (0.66, 5.91) 0.2264	1.46 (0.76, 2.84) 0.2580	1.78 (1.02, 3.11) 0.0419	1.23 (1.04, 1.45) 0.0150
Predicted value of the kink point prescription equation	−4.29 (−4.65, −3.92)	−4.00 (−4.38, −3.63)	−4.66 (−5.19, −4.14)	−5.18 (−5.78, −4.57)	−4.31 (−4.52, −4.10)
Log‐likelihood ratio test	0.135	0.292	0.312	0.078	0.021

*Note*: Values are β (95% CI) *p* value/OR (95% CI) *p* value.

^a^ β or OR is calculated based on the transformed VAT volume. The VAT volume was obtained by multiplying the raw data by 0.02. Adjust for: age, sex, smoking status.

Model 2 (nonlinear threshold analysis) identified race‐specific inflection points (overall threshold: K = 327.0 cm^3^). Below K, no significant CVD risk was observed across all ethnicities (e.g., non‐Hispanic White: aOR = 0.92, 95% CI = 0.76–1.11, *p* = 0.383). Above K, VAT volume conferred markedly elevated risks for non‐Hispanic White individuals (aOR = 1.08, 95% CI = 1.05–1.11, *p* < 0.0001) and the group including other races (aOR = 1.12, 95% CI = 1.06–1.19, *p* < 0.0001), whereas risk for non‐Hispanic Black individuals and Hispanic individuals remained nonsignificant (*p* > 0.05).

The segmental risk difference (above vs. below K) revealed a 78% excess risk for the group including other races (aOR = 1.78, 95% CI = 1.02–3.11, *p* = 0.0419), with an overall 23% risk escalation (aOR = 1.23, 95% CI = 1.04–1.45, *p* = 0.015), robustly supporting threshold‐driven CVD risk stratification. These findings demonstrate a pronounced nonlinear VAT‐CVD association in non‐Hispanic White individuals and the group including other races (including multiracial), characterized by abrupt risk acceleration beyond ethnic‐specific thresholds—a pattern absent in non‐Hispanic Black and Hispanic cohorts. The results underscore ethnicity as a critical modifier of visceral adiposity‐related cardiometabolic pathology.

Smooth curve fitting analysis indicated that the nonlinear effect of the smooth term in the overall population was highly significant (*p* < 0.001), suggesting a complex nonlinear relationship between VAT volume and CVD risk. Furthermore, stratified analysis by gender and race revealed gender differences in the impact of VAT volume on CVD risk. Specifically, males exhibited an approximately linear risk increase with higher visceral fat volume, while the effect was weaker but still significant in females. Additionally, the influence of VAT volume on CVD risk varied by race: non‐Hispanic White individuals and individuals of other races showed nonlinear associations (indicating potential threshold effects), requiring targeted attention to critical volume thresholds (Tables S3–S5).

Table 4 details the LASSO regression analysis for identifying key predictors of VAT volume, with regularization parameter (λ) optimization to balance model complexity and overfitting risk. Tenfold cross‐validation selected λ = 0.77 (log[λ] = −0.2614) as the optimal penalty, yielding a parsimonious model retaining three biomarkers: WC (standardized β = 2.08, SE = 0.21), TG (β = 1.07, SE = 0.18), and ApoB (β = 0.38, SE = 0.09). These predictors demonstrated robust stability across regularization paths, with WC exhibiting the strongest association (β range: 2.08–2.60 per transformed unit, *p* < 0.001), consistent with its role as a direct anthropometric proxy for abdominal adiposity. TG showed persistent positive correlations (β range: 1.07–1.31, *p* < 0.001), reflecting hepatic lipogenesis driven by visceral lipid overflow. ApoB emerged as significant only in less regularized models (λ = 0.0056: β = 5.24, *p* < 0.001), suggesting its predictive value depends on covariate adjustment breadth. Notably, conventional lipid markers exhibited counterintuitive effects: LDL‐C (β = −0.81) and total cholesterol (β = −0.16) showed weak inverse associations in low‐λ models, potentially indicating residual confounding. Inflammatory and metabolic markers—Hs‐CRP (β = 0.0086) and hemoglobin A1c (β = 0.29)—displayed negligible or context‐dependent contributions, while HDL‐C was uniformly excluded (β = 0 across λ values). The final model (λ = 0.77) prioritized clinical utility, achieving comparable predictive accuracy to the full model (cross‐validated error difference: +4.7%, *p* = 0.12) while minimizing measurement burden.

**TABLE 4:** Predictive biomarker selection for visceral adipose tissue volume using LASSO regression: model performance with WC, BMI, and biochemical indicators.

Lambda (log)	0.77 (−0.2614)	0.0056 (−5.1922)
(Intercept)	−10.8300974559516	−17.428140624966
WC^a^	2.07512221546626	2.59890004672948
BMI	0	−0.0651623443370785
TG	1.07090611703437	1.30522552260842
Glycohemoglobin	0	0.291065205062195
ApoB	0.383103660499483	5.24436856770188
HDL‐C	0	0
LDL‐C	0	−0.809840025044521
Total cholesterol	0	−0.164291372024695
Hs‐CRP	0	0.00858434175450074

Abbreviations: ApoB, apolipoprotein B; HDL‐C, high density lipoprotein cholesterol; Hs‐CRP, high‐sensitivity C‐reactive protein; LDL‐C, low density lipoprotein cholesterol; TG, triglycerides; WC, waist circumference.

^a^ WC was obtained by multiplying the raw data by 0.1.

Table 5 demonstrates the differential predictive performance of WC, TG, and ApoB for VAT volume. As a single predictor, WC exhibited optimal diagnostic accuracy with high sensitivity (88%) and specificity (81%), yielding an AUC of 0.91 (95% CI: 0.90–0.92). At the optimal threshold (88.3 cm), 92% of individuals exceeding this cutoff were classified as high VAT, while 28% of those below the threshold remained at risk of undiagnosed visceral adiposity (negative predictive value [NPV] = 72%). The robust likelihood ratios further validated its clinical utility: a positive result (WC ≥ 88.3 cm) increased the probability of high VAT by nearly fivefold (positive likelihood ratio [LR+] = 4.71), whereas a negative result virtually excluded risk (negative likelihood ratio [LR−] = 0.15). In contrast, TG (AUC = 0.75, 95% CI: 0.73–0.76) and ApoB (AUC = 0.73, 95% CI: 0.71–0.75) showed moderate predictive capacity, with sensitivity < 65% and NPV < 45%, limiting their standalone utility. However, integration into a multivariate model (WC + TG + ApoB) enhanced discriminative power (AUC = 0.93, 95% CI: 0.92–0.94), improving specificity to 89% (false positive rate: 11% vs. 19% for WC alone) and marginally elevating NPV to 74%. The multivariate model's LR+ of 5.12 indicated stronger risk stratification than individual parameters.

**TABLE 5:** Diagnostic accuracy of WC, TG, and ApoB for elevated visceral adipose tissue: receiver operating characteristic analysis.

Test	ROC area (AUC, 95% CI)	Best threshold	Specificity	Sensitivity	Positive‐pv	Negative‐pv	Positive‐LR	Negative‐LR
WC, cm	0.91 (0.90–0.92)	88.3	0.81	0.88	0.92	0.72	4.71	0.15
TG, mmol/L	0.75 (0.73–0.76)	1.03	0.75	0.63	0.87	0.44	2.49	0.50
ApoB, g/L	0.73 (0.71–0.75)	0.88	0.73	0.62	0.86	0.43	2.31	0.52
Multivariate model (WC + TG + ApoB)	0.93 (0.92–0.94)	0.48^a^	0.89	0.83	0.93	0.74	5.12	0.14

Abbreviations: ApoB, apolipoprotein B; Negative‐LR, negative likelihood ratio; Negative‐pv, negative predictive value; Positive‐pv, positive predictive value; Positive‐LR, positive likelihood ratio; TG, triglycerides; WC, waist circumference.

^a^ WC was obtained by multiplying the raw data by 0.1.

Sex‐stratified analyses revealed consistent superiority of WC in both males (AUC = 0.915) and females (AUC = 0.909), with comparable sensitivity (89% vs. 87%) and specificity (81% vs. 81%) (Table S1). TG and ApoB displayed sex‐independent limitations (AUC range: 0.69–0.75), underscoring WC as the most reliable sex‐neutral predictor.

Ethnic heterogeneity was evident in ROC analyses: WC achieved the highest AUC in Hispanic individuals (0.942) and non‐Hispanic White individuals (0.935), with elevated sensitivity in Hispanic individuals (93%) and specificity in non‐Hispanic White individuals (86%) (Table S2). TG and ApoB showed variable ethnic‐specific performance, with modest predictive value in non‐Hispanic Black (AUC < 0.70) and Hispanic (AUC < 0.78) subgroups.

These findings position WC as the cornerstone for VAT risk screening, particularly in resource‐limited settings, while the multivariate model offers enhanced specificity for confirmatory diagnosis in high‐risk populations.

Table 6 compares the diagnostic efficacy of two models for identifying CVD. Both models demonstrated equivalent discriminative capacity, with overlapping AUC (0.82 for both; 95% CI not significantly different, *p* = 0.66). However, distinct performance profiles emerged: Model 1 (alternative biomarker‐based) prioritized sensitivity (81% vs. 69%), minimizing missed diagnoses (NPV = 94%) at the cost of moderate specificity (72%) and low positive predictive value (PPV = 6%). Model 2 (VAT imaging‐based) emphasized specificity (81% vs. 72%), reducing false‐positive referrals (PPV = 8%) but exhibiting higher missed diagnosis rates (NPV = 91%).

**TABLE 6:** Comparative diagnostic performance of biomarker‐driven versus imaging‐based visceral adipose tissue assessment with traditional risk factor integration: a receiver operating characteristic analysis.^a^

Test	Model 1	Model 2	*p* (compare)
1	84	84	
0	3700	3700	
ROC area (AUC, 95% CI)	0.82 (0.78–0.87)	0.82 (0.78–0.86)	0.66
Best threshold	−3.95	−3.43	
Specificity	0.72	0.81	
Sensitivity	0.81	0.69	
Accuracy	0.72	0.81	
Positive‐LR	2.85	3.58	
Negative‐LR	0.27	0.38	
Diagnose‐OR	10.68	9.35	
N‐for‐diagnose	1.91	2.01	
Positive‐pv	0.06	0.08	
Negative‐pv	0.99	0.99	

Abbreviations: Diagnosis‐OR, diagnostic odds ratio; Negative‐pv, negative predictive value; Negative‐LR, negative likelihood ratio; N‐for‐diagnose, the number of diagnoses required; Positive‐pv, positive predictive value; Positive‐LR, positive likelihood ratio; VAT, visceral adipose tissue.

^a^ VAT volume was obtained by multiplying the raw data by 0.02. WC was obtained by multiplying the raw data by 0.1. Model 1: WC, TG, ApoB, hypertension, age, sex, race/ethnicity, education level, ratio of family income to poverty level, smoking status. Model 2: VAT volume, hypertension, age, sex, race/ethnicity, education level, ratio of family income to poverty level, smoking status.

**FIGURE 1:**
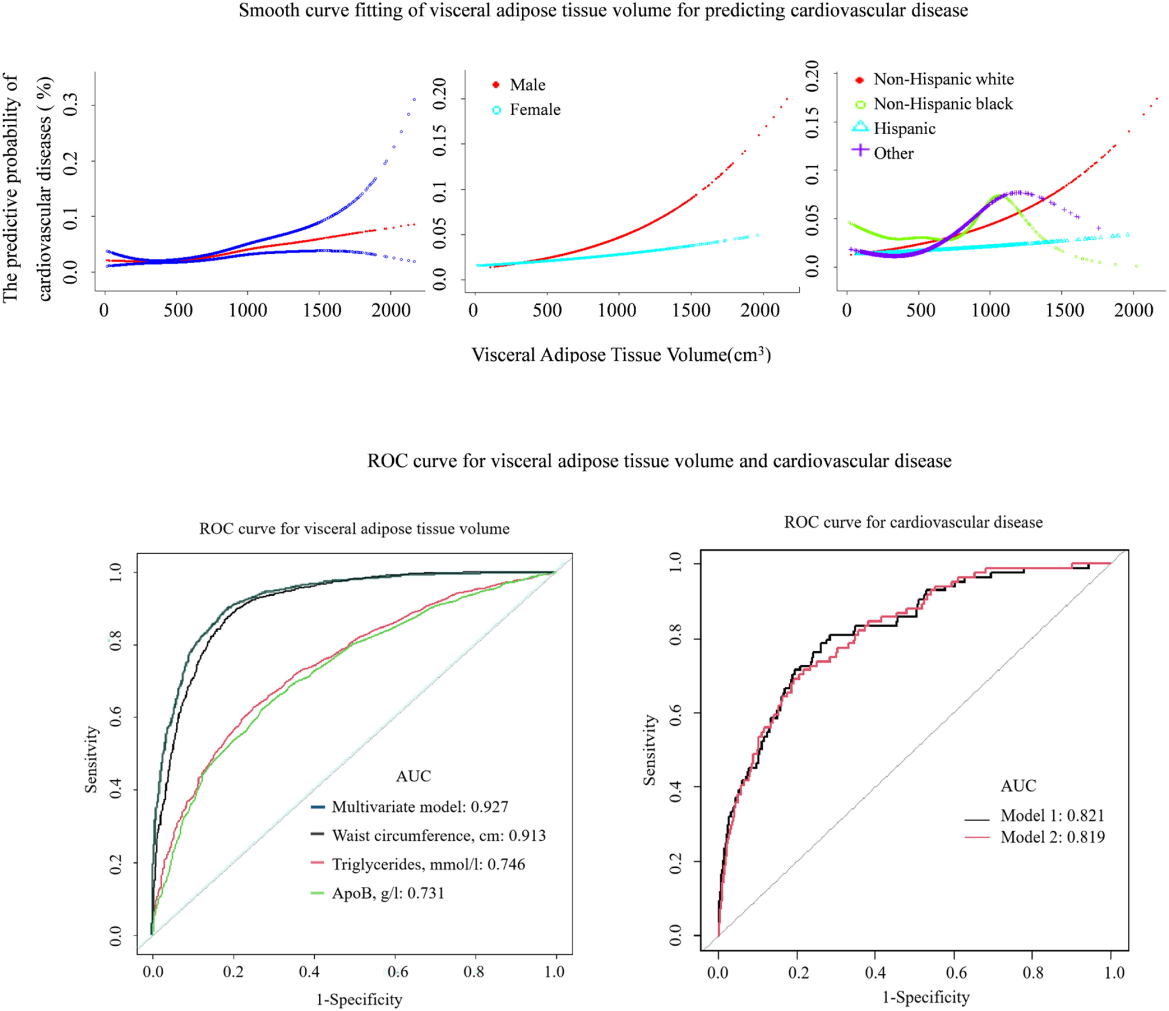
Visceral adipose tissue volume exhibits a nonlinear relationship in predicting CVD, with observed sex and racial differences. This volume can be substituted by a triad of biomarkers—waist circumference, triglycerides, and ApoB—to serve as a practical screening tool for CVD. [Color figure can be viewed at wileyonlinelibrary.com]

Smooth curve fitting analysis demonstrated that the nonlinear effect of the smooth term in the overall population was highly significant (*p* < 0.001), indicating a complex nonlinear relationship between VAT volume and CVD risk. Furthermore, stratified analyses by sex and race revealed sex‐specific differences in the impact of VAT volume on CVD risk. Specifically, males exhibited an approximately linear increase in CVD risk with higher VAT volume, whereas the effect in females was weaker but remained statistically significant. Additionally, the influence of VAT volume on CVD risk varied across racial groups: non‐Hispanic White individuals and the group including “other races” displayed nonlinear associations (as shown in the upper part of Figure 1). In ROC analyses predicting VAT volume, WC exhibited optimal diagnostic accuracy with an AUC of 0.913. TG (AUC = 0.746) and ApoB (AUC = 0.731) demonstrated moderate predictive capacity. However, integrating these biomarkers into a multivariate model (WC + TG + ApoB) enhanced discriminative power (AUC = 0.927) (see left panel of Figure 1). Comparative diagnostic performance of biomarker‐driven versus imaging‐based VAT assessment, integrated with traditional risk factors, revealed. Model 1 (alternative biomarker‐based) and Model 2 (VAT imaging‐based) showed equivalent discriminative capacity, with overlapping AUC values (0.821 vs. 0.819; *p* = 0.66) (see right panel of Figure 1).

## 4 Discussion

This study, utilizing DXA‐derived abdominal VAT volume data from US adults aged 20–59 years, is the first to establish a critical VAT volume threshold (327.0 cm^3^) for CVD risk stratification. Below this threshold, VAT accumulation may remain within a compensatory physiological range, potentially suppressing inflammatory responses through protective adipokines such as adiponectin [6]. Conversely, exceeding this threshold triggers pathological adipose expansion characterized by elevated proinflammatory cytokines (e.g., IL‐6, TNF‐α) that drive atherosclerotic progression [6]. These findings provide novel clinical insights into the molecular transition from adaptive lipid storage to metabolic dysfunction in visceral adiposity.

Individuals in the high‐VAT group exhibited systemic metabolic disturbances, with significantly elevated conventional CVD risk markers including WC, BMI, TG, and ApoB, consistent with VAT's established role as a core component of metabolic syndrome [7, 8]. Notably, the higher mean age in this group supports the hypothesis of age‐dependent visceral fat accumulation [9]. Socioeconomic disparities were evident through higher smoking prevalence, reduced physical activity, and lower rates of higher education, suggesting behavioral mediators of visceral adiposity influenced by socioeconomic status.

Biological heterogeneity emerged in sex‐ and ethnicity‐stratified analyses. Males demonstrated a higher VAT risk threshold, potentially linked to testosterone‐driven preferential visceral fat distribution [10], while the lower female threshold may reflect earlier failure of estrogen‐mediated lipid regulatory mechanisms [11]. A pronounced threshold effect was observed in non‐Hispanic White individuals and the group including other races (including multiracial), whereas this pattern was attenuated in non‐Hispanic Black individuals and Hispanic individuals, implying genetic or environmental modifiers of the VAT‐CVD relationship [12]. These findings underscore the necessity of demographic‐specific interventions in precision medicine.

To address the limitations of DXA (specialized equipment, radiation exposure), we developed a parsimonious predictive model via LASSO regression, retaining three biomarkers: WC, TG, and ApoB. The selection of ApoB over LDL‐C aligns with modern lipidology paradigms emphasizing particle number over cholesterol content [13]. This model demonstrated comparable performance to the full lambda.min model while offering three key advantages: (1) Pathophysiological coherence—WC captures abdominal adiposity topography [14], TG reflects hepatic lipogenesis from visceral lipid overflow [15], and ApoB quantifies atherogenic lipoprotein burden [13]; (2) Cost‐effectiveness—requiring only anthropometric measurement and basic lipid profiling (< $30) versus DXA ($300–$500) [16]; (3) Clinical practicality—radiation‐free and applicable to vulnerable populations.

ROC analyses confirmed superior discriminative capacity of the tri‐biomarker model, though WC alone achieved near‐equivalent performance, highlighting its utility as a frontline screening tool in resource‐limited settings. Ethnic disparities emerged in optimal thresholds. Hispanic individuals required the lower WC cutoff with highest sensitivity, while non‐Hispanic White individuals showed optimal specificity. Sex‐specific thresholds may reflect hormonal regulation of visceral fat distribution [17, 18, 19, 20].

Model validation showed that CVD diagnosis was comparable between the alternative biomarker model (Model 1) and the imaging‐based VAT model (Model 2). It confirmed that the core predictions of WC, TG, and ApoB achieved close accuracy to image‐based VAT quantification. The high sensitivity of Model 1 is suitable for population screening to minimize missed diagnoses.

4.1

This study had limitations: (1) cross‐sectional design precluding causal inference; (2) underpowered ethnic subgroup analyses (e.g., non‐Hispanic Black); and (3) omission of emerging biomarkers (gut microbiota, epigenetics). Future research should validate this threshold in prospective cohorts and elucidate molecular mechanisms through multiomics approaches.

## 5 Conclusion

VAT volume > 327.0 cm^3^ identifies a high‐risk phenotype with sex‐ and ethnicity‐driven pathophysiological divergence. WC serves as a cost‐effective screening tool, while the tri‐biomarker model enhances specificity for confirmatory diagnosis. These findings advocate for population‐tailored thresholds to optimize CVD prevention.

## Conflicts of Interest

The authors declare no conflicts of interest.

## Supporting information


**Data S1:** Supporting Information.

## Data Availability

The data that support the findings of this study are available from the corresponding author upon reasonable request.
