# Supplementary material for: Visceral Adiposity Thresholds for Cardiovascular Risk Stratification: A Simplified Biomarker‐Driven Model
Source: Obesity (Silver Spring). 2025 Aug 18;33(10):2005–13. doi: 10.1002/oby.24367 (PMC12477109; doi:10.1002/oby.24367)
Supplement: Supplementary file 1 — Data S1: Supporting Information. [file OBY-33-2005-s001.docx]

| **Supplemental Table 1 Sex-Specific Diagnostic Accuracy of Waist Circumference, Triglycerides, and Apolipoprotein B for Elevated Visceral Adipose Tissue: Receiver Operating Characteristic Analysis^﹟^** | | | | | | | | | | |
| --- | --- | --- | --- | --- | --- | --- | --- | --- | --- | --- |
| **Test** | ROC area(AUC) | 95%CI low | 95%CI upp | Best threshold | Specificity | Sensitivity | Postive-pv | Negative-pv | Positive-LR | Negative-LR |
| **Waist circumference, cm** | | | | | | | | | | |
| **Male** | 0.9152 | 0.8994 | 0.931 | 88.65 | 0.8136 | 0.8896 | 0.9375 | 0.7012 | 4.7732 | 0.1357 |
| **Female** | 0.9094 | 0.8955 | 0.9234 | 87.25 | 0.8103 | 0.8724 | 0.9082 | 0.7471 | 4.6002 | 0.1574 |
| **Triglycerides, mmol/l** | | | | | | | | | | |
| **Male** | 0.7506 | 0.7273 | 0.7739 | 1.1235 | 0.7628 | 0.6289 | 0.8922 | 0.3971 | 2.6518 | 0.4865 |
| **Female** | 0.7351 | 0.7124 | 0.7578 | 0.8975 | 0.707 | 0.6619 | 0.8296 | 0.4924 | 2.2588 | 0.4783 |
| **ApoB, g/l** | | | | | | | | | | |
| **Male** | 0.7365 | 0.7121 | 0.7609 | 0.865 | 0.6897 | 0.6783 | 0.8721 | 0.4072 | 2.186 | 0.4665 |
| **Female** | 0.7185 | 0.6954 | 0.7416 | 0.835 | 0.6822 | 0.6511 | 0.8153 | 0.4757 | 2.0485 | 0.5115 |
| ﹟ Waist circumference was obtained by multiplying the raw data by 0.1. Positive pv: Positive predictive value; Negative pv: Negative predictive value; Positive-LR: Positive likelihood ratio; Negative-LR: Negative likelihood ratio | | | | | | | | | | |

| **Supplemental Table 2 Racial/Ethnic Heterogeneity in Diagnostic Accuracy of Waist Circumference, Triglycerides, and Apolipoprotein B for Elevated Visceral Adipose Tissue: Receiver Operating Characteristic Analysis^＆^** | | | | | | | | | | |
| --- | --- | --- | --- | --- | --- | --- | --- | --- | --- | --- |
| **Test** | ROC area(AUC) | 95%CI low | 95%CI upp | Best threshold | Specificity | Sensitivity | Postive-pv | Negative-pv | Positive-LR | Negative-LR |
| **Waist circumference, cm** | | | | | | | | | | |
| **Non-Hispanic white** | 0.9346 | 0.9211 | 0.948 | 90.3 | 0.8625 | 0.8637 | 0.946 | 0.6942 | 6.2813 | 0.1581 |
| **Non-Hispanic black** | 0.8769 | 0.8513 | 0.9024 | 89.25 | 0.6949 | 0.9134 | 0.8464 | 0.8135 | 2.9941 | 0.1246 |
| **Hispanic** | 0.942 | 0.9231 | 0.9609 | 87.05 | 0.8324 | 0.9293 | 0.9627 | 0.7163 | 5.5446 | 0.085 |
| **Other** | 0.9326 | 0.9144 | 0.9508 | 85.6 | 0.9125 | 0.8098 | 0.9403 | 0.7385 | 9.2604 | 0.2084 |
| **Triglycerides, mmol/l** | | | | | | | | | | |
| **Non-Hispanic white** | 0.7353 | 0.7086 | 0.762 | 1.067 | 0.7111 | 0.6308 | 0.8584 | 0.4097 | 2.1835 | 0.5192 |
| **Non-Hispanic black** | 0.6923 | 0.6553 | 0.7293 | 0.8865 | 0.7324 | 0.5616 | 0.7949 | 0.4751 | 2.099 | 0.5986 |
| **Hispanic** | 0.7833 | 0.7484 | 0.8183 | 0.8975 | 0.6778 | 0.7646 | 0.9173 | 0.3812 | 2.3728 | 0.3474 |
| **Other** | 0.7759 | 0.741 | 0.8107 | 1.033 | 0.7715 | 0.6836 | 0.8351 | 0.5903 | 2.9923 | 0.4101 |
| **ApoB, g/l** | | | | | | | | | | |
| **Non-Hispanic white** | 0.7312 | 0.7041 | 0.7583 | 0.865 | 0.7136 | 0.6388 | 0.8609 | 0.4158 | 2.2303 | 0.5062 |
| **Non-Hispanic black** | 0.6919 | 0.6554 | 0.7284 | 0.835 | 0.6722 | 0.6467 | 0.7846 | 0.5076 | 1.9732 | 0.5255 |
| **Hispanic** | 0.7753 | 0.7393 | 0.8112 | 0.925 | 0.8556 | 0.5969 | 0.9508 | 0.3124 | 4.1324 | 0.4711 |
| **Other** | 0.7186 | 0.6804 | 0.7569 | 0.835 | 0.6404 | 0.6858 | 0.7635 | 0.5463 | 1.9075 | 0.4905 |
| ＆ Waist circumference was obtained by multiplying the raw data by 0.1. Positive pv: Positive predictive value; Negative pv: Negative predictive value; Positive-LR: Positive likelihood ratio; Negative-LR: Negative likelihood ratio | | | | | | | | | | |

| **Supplemental Table 3 Smooth curve fitting of visceral adipose tissue volume for predicting cardiovascular disease, adjusted for age, gender, race, and smoking** | | | | | | | |
| --- | --- | --- | --- | --- | --- | --- | --- |
| **Linear terms effect** | | | | | | | |
| **Estimate** | Std. Error | z value | Pr(>\|z\|) | exp(est) | 95%CI low | 95%CI upp |  |
| **(Intercept)** | -7.391 | 0.4122 | -17.9302 | 0 | 6e-04 | 3e-04 | 0.0014 |
| **Age** | 0.0732 | 0.0074 | 9.884 | 0 | 1.076 | 1.0605 | 1.0917 |
| **Sex** | -0.0971 | 0.1283 | -0.757 | 0.4491 | 0.9075 | 0.7058 | 1.1668 |
| **factor(Non-Hispanic black)** | 0.473 | 0.1569 | 3.0137 | 0.0026 | 1.6048 | 1.1798 | 2.1828 |
| **factor(Hispanic)** | -0.2398 | 0.1797 | -1.3349 | 0.1819 | 0.7868 | 0.5532 | 1.1189 |
| **factor(Other)** | -0.0436 | 0.2005 | -0.2176 | 0.8278 | 0.9573 | 0.6462 | 1.4182 |
| **smoking** | 0.7462 | 0.1323 | 5.6412 | 0 | 2.1089 | 1.6273 | 2.7331 |
|  |  |  |  |  |  |  |  |
| **Chi-square tests for linear terms** | | | | | | | |
| **df** | Chi.sq | p-value |  |  |  |  |  |
| **Age** | 1 | 97.6944 | 0 |  |  |  |  |
| **Sex** | 1 | 0.573 | 0.4491 |  |  |  |  |
| **factor(Race)** | 3 | 17.0503 | 7e-04 |  |  |  |  |
| **smoking** | 1 | 31.8234 | 0 |  |  |  |  |
|  |  |  |  |  |  |  |  |
| **Approximate significance of smooth terms** | | | | | | | |
| **edf** | Ref.df | Chi.sq | p-value |  |  |  |  |
| **s(visceral adipose tissue volume)** | 3.2865 | 4.1398 | 33.1781 | 0 |  |  |  |
|  |  |  |  |  |  |  |  |
| **Model statistics** | | | | | | | |
| **N:** | 11661 |  |  |  |  |  |  |
| **Adj. r-square:** | 0.0289 |  |  |  |  |  |  |
| **Deviance explained:** | 0.1146 |  |  |  |  |  |  |
| **UBRE score (sp.criterion):** | -0.8028 |  |  |  |  |  |  |
| **Scale estimate:** | 1 |  |  |  |  |  |  |
| **family:** | binomial |  |  |  |  |  |  |
| **link function:** | logit |  |  |  |  |  |  |
|  |  |  |  |  |  |  |  |
| The VAT(visceral adipose tissue) volume was obtained by multiplying the raw data by 0.02. | | | | | | | |

| **Supplemental Table 4 Sex-specific smooth curve fitting of visceral adipose tissue volume for predicting cardiovascular disease, adjusted for age, race, and smoking** | | | | | | | |
| --- | --- | --- | --- | --- | --- | --- | --- |
|  |  |  |  |  |  |  |  |
| **Linear terms effect** | | | | | | | |
| **Estimate** | Std. Error | z value | Pr(>\|z\|) | exp(est) | 95%CI low | 95%CI upp |  |
| **(Intercept)** | -7.5324 | 0.374 | -20.1426 | 0 | 5e-04 | 3e-04 | 0.0011 |
| **factor(female)** | 0.0557 | 0.1432 | 0.3891 | 0.6972 | 1.0573 | 0.7985 | 1.3999 |
| **Age** | 0.0719 | 0.0073 | 9.7968 | 0 | 1.0745 | 1.0592 | 1.0901 |
| **factor(Non-Hispanic black)** | 0.482 | 0.1572 | 3.067 | 0.0022 | 1.6193 | 1.19 | 2.2035 |
| **factor(Hispanic)** | -0.2323 | 0.1793 | -1.2958 | 0.1951 | 0.7927 | 0.5578 | 1.1265 |
| **factor(Other)** | -0.055 | 0.2004 | -0.2743 | 0.7839 | 0.9465 | 0.6391 | 1.4019 |
| **smoking** | 0.7664 | 0.1325 | 5.7847 | 0 | 2.1521 | 1.6599 | 2.7902 |
|  |  |  |  |  |  |  |  |
| **Chi-square tests for linear terms** | | | | | | | |
| **df** | Chi.sq | p-value |  |  |  |  |  |
| **Sex** | 1 | 0.1514 | 0.6972 |  |  |  |  |
| **Age** | 1 | 95.9768 | 0 |  |  |  |  |
| **factor(Race)** | 3 | 17.5667 | 5e-04 |  |  |  |  |
| **smoking** | 1 | 33.4628 | 0 |  |  |  |  |
|  |  |  |  |  |  |  |  |
| **Approximate significance of smooth terms** | | | | | | | |
| **edf** | Ref.df | Chi.sq | p-value |  |  |  |  |
| **s(visceral adipose tissue volume):factor(male)** | 1.0042 | 1.0083 | 31.9587 | 0 |  |  |  |
| **s(visceral adipose tissue volume):factor(female)** | 1.0001 | 1.0001 | 4.6576 | 0.0309 |  |  |  |
|  |  |  |  |  |  |  |  |
| **Model statistics** | | | | | | | |
| **N:** | 11661 |  |  |  |  |  |  |
| **Adj. r-square:** | 0.0289 |  |  |  |  |  |  |
| **Deviance explained:** | 0.1145 |  |  |  |  |  |  |
| **UBRE score (sp.criterion):** | -0.8029 |  |  |  |  |  |  |
| **Scale estimate:** | 1 |  |  |  |  |  |  |
| **family:** | binomial |  |  |  |  |  |  |
| **link function:** | logit |  |  |  |  |  |  |
|  |  |  |  |  |  |  |  |
| The VAT(visceral adipose tissue) volume was obtained by multiplying the raw data by 0.02. | | | | | | | |

| **Supplemental Table 5 Race-specific smooth curve fitting of visceral adipose tissue volume for predicting cardiovascular disease, adjusted for age, sex, and smoking** | | | | | | | |
| --- | --- | --- | --- | --- | --- | --- | --- |
| **Linear terms effect** | | | | | | | |
| **Estimate** | Std. Error | z value | Pr(>\|z\|) | exp(est) | 95%CI low | 95%CI upp |  |
| **(Intercept)** | -7.5155 | 0.4233 | -17.7563 | 0 | 5e-04 | 2e-04 | 0.0012 |
| **factor(Non-Hispanic black)** | 0.564 | 0.1735 | 3.2499 | 0.0012 | 1.7577 | 1.2509 | 2.4698 |
| **factor(Hispanic)** | -0.0514 | 0.2173 | -0.2367 | 0.8129 | 0.9499 | 0.6204 | 1.4543 |
| **factor(Other)** | -0.1267 | 0.2442 | -0.5189 | 0.6038 | 0.881 | 0.5459 | 1.4218 |
| **Age** | 0.0737 | 0.0074 | 9.9633 | 0 | 1.0765 | 1.061 | 1.0922 |
| **Sex** | -0.0735 | 0.1286 | -0.5714 | 0.5678 | 0.9292 | 0.7222 | 1.1955 |
| **smoking** | 0.735 | 0.1325 | 5.5492 | 0 | 2.0855 | 1.6087 | 2.7037 |
|  |  |  |  |  |  |  |  |
| **Chi-square tests for linear terms** | | | | | | | |
| **df** | Chi.sq | p-value |  |  |  |  |  |
| **factor(Race)** | 3 | 16.6891 | 8e-04 |  |  |  |  |
| **Age** | 1 | 99.2677 | 0 |  |  |  |  |
| **Sex** | 1 | 0.3264 | 0.5678 |  |  |  |  |
| **smoking** | 1 | 30.7936 | 0 |  |  |  |  |
|  |  |  |  |  |  |  |  |
| **Approximate significance of smooth terms** | | | | | | | |
| **edf** | Ref.df | Chi.sq | p-value |  |  |  |  |
| **s(visceral adipose tissue volume):factor(Non-Hispanic white)** | 1.0015 | 1.0029 | 23.7246 | 0 |  |  |  |
| **s(visceral adipose tissue volume):factor(Non-Hispanic black)** | 4.6492 | 5.7459 | 8.3886 | 0.1661 |  |  |  |
| **s(visceral adipose tissue volume):factor(Hispanic)** | 1.0037 | 1.0073 | 1.1386 | 0.2875 |  |  |  |
| **s(visceral adipose tissue volume):factor(Other)** | 3.228 | 4.0566 | 16.2626 | 0.003 |  |  |  |
|  |  |  |  |  |  |  |  |
| **Model statistics** | | | | | | | |
| **N:** | 11661 |  |  |  |  |  |  |
| **Adj. r-square:** | 0.0321 |  |  |  |  |  |  |
| **Deviance explained:** | 0.1215 |  |  |  |  |  |  |
| **UBRE score (sp.criterion):** | -0.8031 |  |  |  |  |  |  |
| **Scale estimate:** | 1 |  |  |  |  |  |  |
| **family:** | binomial |  |  |  |  |  |  |
| **link function:** | logit |  |  |  |  |  |  |
|  |  |  |  |  |  |  |  |
| The VAT(visceral adipose tissue) volume was obtained by multiplying the raw data by 0.02. | | | | | | | |
